# Advancements in 3D Reconstruction for Plant Phenotyping: Technologies, Applications, Challenges, and Future Directions

**DOI:** 10.3390/s26092730

**Published:** 2026-04-28

**Authors:** Partho Ghose, Al Bashir, Azlan Zahid

**Affiliations:** Department of Biological and Agricultural Engineering, Texas A&M AgriLife Research, Texas A&M University System, Dallas, TX 75252, USA; partho.ghose@tamu.edu (P.G.); albashir@tamu.edu (A.B.)

**Keywords:** sensors, 3D reconstruction, deep learning, plant phenotyping

## Abstract

Recent advancements in 3D reconstruction technologies have significantly transformed plant phenotyping, enabling precise, scalable, and automated trait extraction. Traditional manual phenotyping methods are increasingly being replaced by image-based approaches, such as photogrammetry, LiDAR, RGB-D sensing, and deep learning (DL)-based techniques. These tools allow for non-destructive, high-throughput measurements of plant morphology, structure, and physiological traits. This review synthesizes the state of the art in 3D reconstruction methods, including conventional geometric algorithms and emerging DL methods, and evaluates their application across diverse plant species. In addition, we discuss the sensing modalities, evaluation metrics, and crop-specific deployments. Although promising, current technologies still face challenges in terms of computational efficiency, scalability to outdoor environments, and generalizability across crop types. This review concludes by identifying research gaps and future directions for making real-time, field-deployable 3D phenotyping systems.

## 1. Introduction

Plant phenotyping involves the process of quantifying plant traits such as morphology, physiology, and growth dynamics [1,2]. Accurate phenotyping enables plant breeders and researchers to identify genotypes with desirable traits, contributing to the development of higher-yielding, stress-tolerant, and resource-efficient crops [3]. Traditional plant phenotyping relies on manual observation and measurement, making it time-consuming and labor-intensive [1,2]. As agricultural systems are expanding and diversifying, these conventional approaches no longer meet the throughput and precision required for large-scale phenotyping programs [3]. In recent years, imaging-based methods have offered scalable alternatives to capture surface-level morphological traits such as leaf size, shape, and color [4]. However, 2D imaging remains insufficient for traits that require spatial context- such as canopy structure, leaf angle, and plant volume, leading to inaccuracies in trait estimation and limited insights into plant–environment interactions [3]. To overcome these limitations, the integration of 3D reconstruction technologies into plant phenotyping has gained momentum. This enables the generation of high-resolution spatial plant models, facilitating precise trait quantification and time-series analysis under both controlled and field conditions [3,4,5]. 3D reconstruction procedures are broadly categorized into active sensing techniques, such as Light Detection and Ranging (LiDAR), RGB-D, and structured light systems, which provide direct depth information through laser or infrared projection. On the other hand, passive techniques, such as multi-view stereo (MVS) and structure-from-motion (SfM), estimate depth through photogrammetric computation of images captured from different viewpoints [6,7]. Among active sensing methods, LiDAR has shown strong performance in capturing complex canopy structures, especially in outdoor conditions due to its robustness to lighting variations and occlusions [8]. RGB-depth (RGB-D) cameras combine color and depth sensing in a compact form, enabling rapid image acquisition in controlled settings [9]. While these technologies have improved the resolution and throughput of phenotyping, they still face challenges including noisy point clouds, incomplete reconstructions, and computational bottlenecks, particularly when applied to densely vegetated or occluded environments [10,11]. In response to these limitations, recent advancements in deep learning-based 3D reconstruction, particularly Neural Radiance Fields (NeRF) [12] and 3D Gaussian Splatting (3DGS) [12], offer a promising new direction. These neural implicit models reconstruct highly detailed 3D geometry and appearance from sparse multi-view images without requiring depth supervision, making them suitable for highly occluded plant structures [12,13]. NeRF encodes radiance and density fields through volumetric rendering, achieving photorealistic synthesis from novel viewpoints. Similarly, 3DGS provides fast and scalable rendering while retaining explicit geometric properties. These techniques are especially valuable in phenotyping contexts where precision, automation, and adaptability are essential. Furthermore, recent studies have begun to explore integrating AI-driven 3D reconstruction into plant phenotyping workflows. For example, a novel framework has been proposed to enhance point cloud segmentation using deep neural networks (NNs) [14], align multi-sensor data streams with minimal user input [15], and extract phenotypic traits automatically from reconstructed models [16]. These innovations promise to reshape plant phenotyping by improving the resolution, scalability, and interpretability of trait measurements. Despite notable progress in plant phenotyping research, a critical assessment of the current literature reveals several persisting limitations. Previous reviews have primarily concentrated on traditional image-based methods and the general applications of computer vision in agriculture, without explicit focus on 3D reconstruction frameworks [17,18,19,20,21]. Although recent works have begun to explore 3D imaging modalities [22,23], these efforts are often fragmented, rarely encompassing advanced neural reconstruction paradigms such as NeRF or 3DGS (Table 1). Furthermore, critical topics such as the synthesis of the state-of-the-art 3D reconstruction systems, benchmarking with standardized evaluation metrics, and crop-specific 3D reconstruction remain underexplored [24,25]. Despite rapid progress in 3D phenotyping, the literature remains fragmented across different sensing modalities, reconstruction paradigms, and crop-specific implementations. In particular, it is still unclear when classical geometry-based pipelines are sufficient, when neural rendering methods such as NeRF and 3D Gaussian Splatting provide clear advantages, and what practical barriers still limit their translation to field-scale phenotyping. To date, no comprehensive review has systematically synthesized classical methods, deep learning advancements, performance metrics, and publicly available software for 3D plant phenotyping. This review addresses this gap by synthesizing active and passive sensing systems, conventional and deep learning-based reconstruction strategies, evaluation criteria, and crop-wise applications within a single comparative framework, as given in Table 1. Rather than only cataloguing prior work, we highlight the trade-offs among geometric fidelity, rendering realism, computational cost, sensing complexity, and downstream trait utility, and identify the methodological priorities needed for robust, scalable, and field-deployable 3D plant phenotyping.

## 2. Article Selection

**Figure 1:**
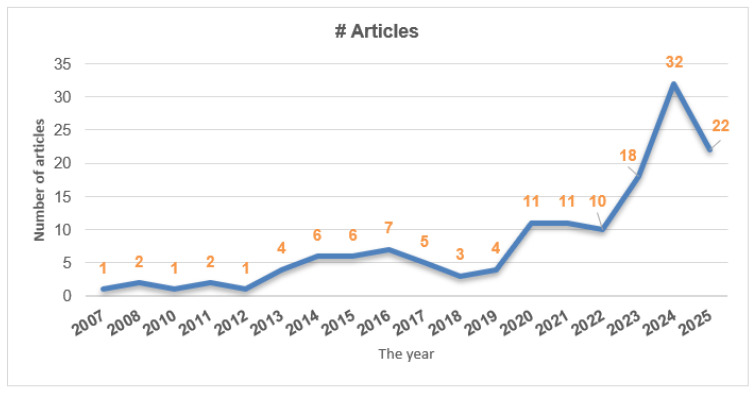
Year-wise selected number of articles.

As shown in Figure 1, a total of 148 articles were selected for this review, spanning the years 2007 to 2025. Only one to two articles were selected from the earlier years (2007–2012), while the number gradually increased to 4–7 articles between 2013 and 2019. From 2020 onwards, 11 articles were selected from 2020 and 2021 each, followed by 10 in 2022, and a notable peak of 32 articles in 2024 for inclusion in the review. For the most recent year, 2025, 22 articles were selected, reflecting the growing volume of relevant research in recent years. Among the listed sources, 32 publications were collected from Elsevier, accounting for the largest share. From the IEEE, 29 publications were selected, indicating strong representation in engineering and technology domains. From Springer and MDPI, 18 and 17 manuscripts were collected, respectively, while Science (7), Cell (3), Nature (1), and ASABE (1) had comparatively smaller portions. Other publishers, grouped under “Others,” account for 22 publications. We also selected research articles from Frontiers (6), ACM (6), and Wiley (5); finally, after screening, 139 articles were kept for the final review.

## 3. Techniques for 3D Reconstruction

**Figure 2:**
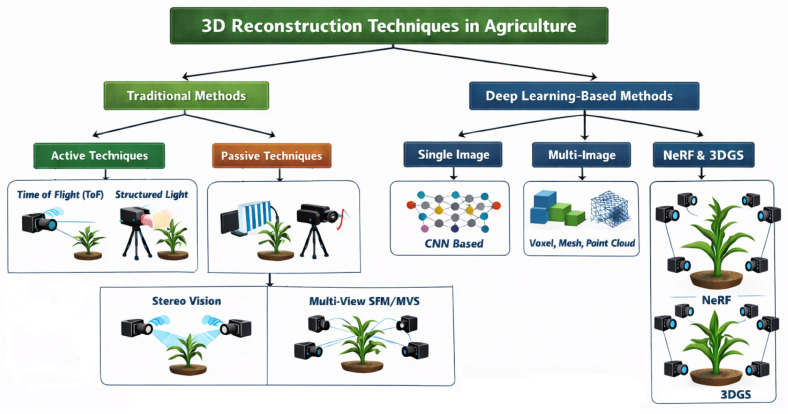
GenAI in precision agriculture and decision support.

The 3D reconstruction is categorized into two main approaches: traditional and deep learning-based methods [27]. Traditional techniques are further categorized into active and passive types [28] and typically rely on geometric algorithms, camera calibration, and manual feature engineering [28]. In contrast, DL methods use NNs to automatically learn features and map 2D inputs to 3D outputs, offering improved adaptability and eliminating the need for handcrafted designs, as illustrated in Figure 2.

### 3.1. Traditional Methods

Classical active methods for 3D reconstruction estimate depth by emitting light or infrared signals and analyzing their reflections [29]. Key approaches include time-of-flight (ToF), triangulation, and structured light. ToF sensors generate depth maps in a single exposure, enabling low-cost, real-time plant monitoring under low-light conditions [30,31]. However, their accuracy is highly sensitive to ambient illumination and long-range measurements [32]; nevertheless, they remain effective for field monitoring and growth tracking [32,33]. Triangulation computes depth by forming a geometric relationship between the projector, sensor, and target [34]. Although conceptually simple, incremental Delaunay triangulation is significantly slower on large datasets, motivating improvements such as KD-tree indexing [35] and Hilbert curve ordering [36]. Its close-range accuracy is strong but degrades at longer distances owing to field-of-view and resolution limitations [37]. Structured light projects coded patterns and reconstructs surfaces from the observed distortions [38]. The accuracy of the system declines under bright lighting or extended ranges [39]. Methods include triangulation-based projections and phase-based techniques, such as Fourier Transform Profilometry and Phase Measurement Profilometry [40]. Recent multifrequency heterodyne phase fusion has reduced saturation and phase-jump artifacts, yielding smoother high-resolution surfaces [41]. Passive 3D reconstruction infers geometry from images without emitted signals, making it suitable for reflective, transparent, and low-texture plant surfaces [42,43]. Stereo vision estimates depth from the disparities between calibrated cameras through feature detection, matching, and triangulation [44,45]. It is low-cost and effective for dynamic scenes but fails under low illumination or weak textures [46]. Hardware accelerators, such as FPGAs and GPUs, now achieve 20–30 fps, improving real-time viability [47,48]. Multiview vision generalizes stereo to multiple viewpoints and forms the basis of structure-from-motion (SfM) and multi-view stereo (MVS) pipelines [49,50,51,52]. SfM estimates camera poses and generates sparse point clouds using features such as Scale Invariant Feature Transform (SIFT), Oriented Fast and Rotated BRIEF (ORB) or Speeded Up Robust Feature (SURF) [53,54], whereas MVS densifies them into detailed reconstructions [55,56]. Combined SfM–MVS workflows integrate feature extraction, pose estimation, bundle adjustment, and dense stereo matching [57]. However, classical techniques struggle to precisely reconstruct scenes that involve challenging lighting, reflective materials, or surfaces lacking texture. This is mainly because the point clouds generated are sparse and rely heavily on features extracted from two-dimensional images.

### 3.2. Deep Learning-Based Methods

3D reconstruction in modern DL-based methods may differ according to the input type and shape representation [27]. Single-image approaches use end-to-end networks to generate voxels, point clouds, or implicit functions that are later converted to meshes, but often yield incomplete models [24]. Multi-image methods overcome this limitation by integrating information across multiple views to achieve higher accuracy. Shape representations include voxels processed with encoder–decoder networks, point clouds consisting of unordered 3D points requiring regularization, and then meshes that capture detailed geometry through vertices, edges, and polygons [58]. Common NNs use CNNs, suitable for 2D image data; RNNs, effective at capturing sequential features; and GCNs, which handle non-Euclidean data structures [27]. Moreover, several deep learning models have also been developed for 3D point cloud generation, including ShapeNet [59], ObjectNet3D [60], Pascal 3D+ [61], and KITTI [62], which are primarily trained on large-scale benchmark datasets. Although these models have demonstrated strong performance in general object reconstruction, recent research in plant phenotyping has increasingly adopted more advanced implicit and differentiable 3D representations, notably NeRF and 3DGS. These methods offer improved geometric fidelity and photorealistic reconstruction of complex structures. The following subsections provide a concise overview of these approaches.

#### 3.2.1. NeRF

NeRF is an innovative technique designed to generate highly realistic 3D views of objects by learning a continuous volumetric representation of a scene [12]. Unlike traditional methods that explicitly reconstruct 3D models, NeRF uses an NN to represent the scene as a function that maps 3D coordinates and viewing directions to color and density values. This allows the model to produce new views of the object from any angle, making it particularly useful for applications like plant phenotyping [12].

**Figure 3:**
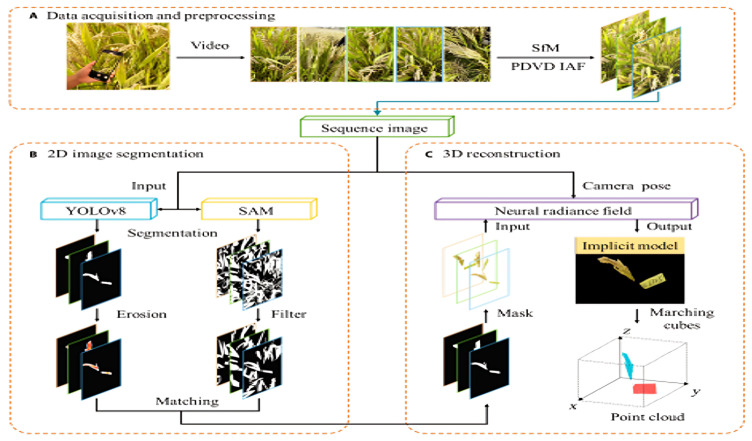
NeRF-based architecture for 3D reconstruction of rice panicle and trait extraction. Adapted from [63].

**Figure 4:**
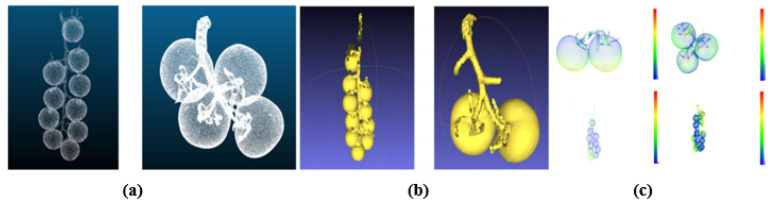
The above image shows the effectiveness of the NeRF model for 3D reconstruction. (**a**) A real tomato model obtained by the scanner; (**b**) predicted tomato plant by the NeRF model and; (**c**) error distribution plot of the tomato point cloud shown using color encoding as shown in [63].

To train a NeRF, multiple images of the same scene are captured from different viewpoints. These images provide the data needed to optimize the NN so that it accurately models how light interacts with the scene at various spatial points and viewing directions [25]. It estimates the color and density for points sampled along rays that pass through the camera’s viewpoint, enabling the synthesis of photorealistic images from novel perspectives. NeRF uses a hierarchical structure in its NN [25]. It first predicts the density based on spatial location alone, then incorporates viewing direction to determine the color. This design ensures consistent rendering across different views. Once trained, the model can also infer depth information, effectively capturing the 3D geometry of the scene as shown in Figure 3. By comparing the synthesized images to the original input images during training, the network continuously improves its accuracy, resulting in detailed and realistic 3D reconstructions as shown in Figure 4.

In summary, NeRF demonstrates notable advantages in reconstructing plant structures under sparse viewpoint conditions due to its ability to learn continuous volumetric representations from limited multi-view images. This makes it particularly effective in scenarios where dense image acquisition is impractical. Additionally, NeRF inherently models view-dependent radiance, allowing it to better handle illumination variations and produce photorealistic renderings under changing lighting conditions. However, its performance degrades in highly occluded plant canopies, where dense foliage limits visibility and reduces the accuracy of inferred geometry. Furthermore, NeRF often struggles with thin and repetitive structures, such as stems and fine leaf edges, leading to blurred or incomplete reconstructions. These limitations highlight the need for improved sampling strategies or hybrid approaches when applying NeRF to complex agricultural environments.

#### 3.2.2. 3DGS

**Figure 5:**
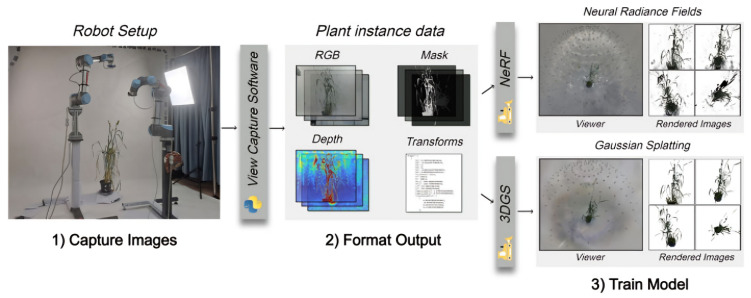
A typical 3D reconstruction pipeline starts by capturing images with their camera poses. The data is then preprocessed—refining poses via bundle adjustment and generating masks as needed. Finally, the formatted dataset is used to train reconstruction models such as NeRF and 3D Gaussian Splatting in NeRFStudio (taken from [65]).

3D Gaussian Splatting (3DGS) overcomes NeRF’s limitations, such as high computational costs and long training times, by enabling real-time rendering, accurate reconstruction, and explicit geometric modeling [64]. Unlike the implicit radiance fields of NeRF, 3DGS represents scenes as collections of learnable 3D Gaussians parameterized by position, covariance (decomposed into rotation and scaling), color, and opacity, with view-dependent effects modeled via spherical harmonics [25]. Rendering is performed by splatting Gaussians onto the image plane, where α-blending combines their contributions based on the opacity and distance as shown in Figure 5 [65]. Frustum culling, tile-based rasterization, and CUDA acceleration improve efficiency, making 3DGS faster than the ray marching of NeRF [64,66]. Training typically initializes Gaussians from the SfM point clouds and optimizes the parameters to align the rendered and ground-truth views. Dynamic densification and pruning refine Gaussian distributions using gradient and opacity cues [25]. The loss functions combine L1 color reconstruction with D-SSIM for balanced photometric and structural accuracy. This framework delivers high-quality reconstructions while drastically reducing the training and inference times compared with NeRF, as detailed in [65].

3DGS offers significant advantages in terms of rendering speed and computational efficiency, enabling near real-time reconstruction compared to volumetric methods such as NeRF. Its explicit representation using Gaussian primitives allows fast rasterization and scalable processing for large datasets. However, this efficiency introduces trade-offs in geometric fidelity, particularly for plant phenotyping tasks involving thin and intricate structures. The Gaussian representation tends to produce over-smoothing effects, leading to loss of fine details in elements such as stems, leaf edges, and awns. Additionally, rendering artifacts may arise in regions with insufficient Gaussian density or under complex occlusions, resulting in blurred boundaries or incomplete structures. While increasing the number of Gaussians can partially mitigate these issues, it also increases memory usage and reduces computational gains. This highlights a fundamental trade-off between reconstruction speed and fine-scale geometric accuracy in 3DGS-based approaches.

### 3.3. Synthesis for 3D Reconstruction Algorithms

Table 2 compares key algorithms for 3D reconstruction, highlighting their input data types, strengths, and weaknesses. Traditional approaches, such as SfM and MVS excel in generating accurate point clouds but are computationally intensive and sensitive to image quality [67,68]. Depth-based methods, including ToF cameras and LiDAR, offer real-time and high-precision scans but face limitations such as ambient light interference and high costs [32,69]. Emerging neural approaches have further transformed the field. NeRF captures fine details but is computationally demanding [70], while 3DGS offers faster processing but lacks maturity for finer details [66]. Traditional methods like Voxel Carving and Marching Cubes remain useful for basic structures, despite limitations with precision and noise [71]. DL models, such as CNNs and PointNet, are robust for handling complex shapes but require large, annotated datasets [27,72]. Hybrid approaches combining SfM and MVS improve reconstruction accuracy by leveraging both methods’ strengths, but increasing computational complexity [73]. These advancements cater to the growing demands of 3D reconstruction across various domains.

However, in the reviewed literature, no single reconstruction family is universally optimal for plant phenotyping. Classical SfM/MVS pipelines remain attractive when metric point clouds, mature software support, and relatively modest computational cost are the main priorities, especially in controlled environments with stable illumination and sufficient texture. Active sensors such as LiDAR and RGB-D cameras further improve depth reliability and throughput, but they may sacrifice portability, cost-efficiency, or fine organ detail depending on scene scale and hardware quality. By contrast, NeRF and 3DGS are especially promising for plants with severe self-occlusion and visually complex canopies because they model view-dependent appearance more effectively and can generate dense scene representations from multi-view imagery. However, their apparent visual realism should not be conflated with phenotype accuracy: thin organs, texture-poor stems, wind-induced motion, and outdoor illumination changes remain difficult, and trait-level validation is still less standardized than in conventional point-cloud workflows.

### 3.4. Evaluation Criteria for 3D Reconstruction

#### 3.4.1. Pixel-Level Evaluation Metrics

Pixel-level metrics are used to evaluate the quality of 2D images rendered from reconstructed 3D models by directly comparing them with ground truth images. Peak Signal-to-Noise Ratio (PSNR): PSNR is a logarithmic measure that quantifies how much a reconstructed image differs from the reference (ground truth) image, based on the Mean Squared Error (MSE). A higher PSNR value indicates better reconstruction quality, meaning the reconstructed textures and surface features closely match the plant’s actual appearance. The PSNR is calculated as:

(1) $$\mathrm{PSNR}=10\times \log_{10}\left(\frac{MAX_{I}^{2}}{\mathrm{MSE}}\right)$$

where $MAX_{I}$ is the highest possible pixel value of the image (e.g., 255 for an 8-bit image), MSE is the mean squared error between the reconstructed and the original image. In the context of 3D plant modeling, PSNR is particularly useful for evaluating how well the reconstruction retains surface textures and fine visual details [75].

**Structural Similarity Index (SSIM):** SSIM is a perceptual metric that assesses the visual similarity between a reconstructed image and its reference counterpart. Unlike pixel-wise error measures, SSIM evaluates image quality by simultaneously comparing three components: brightness (luminance), contrast, and structural patterns. It is particularly valuable in determining whether the reconstructed image maintains the structural integrity and overall visual realism of the original scene. The SSIM between two images *x* and *y* is calculated as:

(2) $$\mathrm{SSIM}\left(x,y\right)=\frac{\left(2\mu_{x}\mu_{y}+C_{1}\right)\left(2\sigma_{xy}+C_{2}\right)}{\left(\mu_{x}^{2}+\mu_{y}^{2}+C_{1}\right)\left(\sigma_{x}^{2}+\sigma_{y}^{2}+C_{2}\right)}$$

where $\mu_{x}$ and $\mu_{y}$ are the average pixel values (means) of images *x* and *y*, $\sigma_{x}^{2}$ and $\sigma_{y}^{2}$ represent their variances, $\sigma_{xy}$ is the covariance between *x* and *y*, $C_{1}$ and $C_{2}$ are small constants to stabilize the calculation when the denominator is close to zero. Higher SSIM values (closer to 1) indicate that the reconstructed image better preserves the visual structure and contrast of the original. This makes SSIM a preferred choice in 3D plant reconstruction for evaluating surface consistency and realism [25].

**Learned Perceptual Image Patch Similarity (LPIPS):** LPIPS is a perceptual similarity metric that compares the visual appearance of two images by analyzing deep feature representations extracted from a trained NN. Instead of comparing raw pixels, LPIPS measures the distance between high-level features across multiple layers, which better aligns with human visual perception. The LPIPS score between images *x* and *y* is computed as:

(3) $$\mathrm{LPIPS}\left(x,y\right)=\sum_{l}\frac{1}{H_{l}W_{l}}\sum_{h,w}{∥w_{l}⊙\left({\hat{x}}_{hw}^{l}-{\hat{y}}_{hw}^{l}\right)∥}_{2}^{2}$$

where ${\hat{x}}_{hw}^{l}$ and ${\hat{y}}_{hw}^{l}$ are the normalized feature activations at layer *l*, location (h,w) from a deep network, $w_{l}$ represents learned weights applied at each layer, $H_{l}$ and $W_{l}$ denote the height and width of the feature maps at layer *l*, ⊙ indicates element-wise multiplication, ${∥\cdot ∥}_{2}^{2}$ is the squared L2 norm. Lower LPIPS values indicate that the compared images are more visually similar in terms of perceived structure and texture. This metric is especially effective for evaluating how natural and realistic reconstructed plant images appear [25].

#### 3.4.2. Geometry-Level Evaluation Metrics

Geometry-level metrics assess how accurately a reconstructed 3D model captures the true shape and structure of the original object. These metrics play a crucial role in verifying that the model preserves both the correct morphology and the overall structural details.

**Intersection over Union (IoU):** IoU evaluates the extent of overlap between a predicted segmentation mask *F* and the ground truth Γ. It measures how well the reconstructed structure corresponds to the actual reference by taking the ratio of their intersection over their union:

(4) $$\mathrm{IoU}=\frac{\left|F\cap \Gamma \right|}{\left|F\cup \Gamma \right|}$$

A higher IoU value indicates more accurate reconstruction and stronger geometric consistency [25,76].

**Chamfer Distance (CD):** Measures the difference between two 3D point clouds by averaging the closest-point distances in both directions. Given a reconstructed point cloud $\Sigma_{1}$ and a ground truth point cloud $\Sigma_{2}$, CD is defined as:

(5) $$\mathrm{CD}\left(\Sigma_{1},\Sigma_{2}\right)=\frac{1}{|\Sigma_{1}|}\sum_{x\in \Sigma_{1}}\min_{y\in \Sigma_{2}}{∥x-y∥}_{2}^{2}+\frac{1}{|\Sigma_{2}|}\sum_{y\in \Sigma_{2}}\min_{x\in \Sigma_{1}}{∥x-y∥}_{2}^{2}$$

Smaller values of CD imply that the reconstructed and reference point clouds are closely aligned, reflecting higher geometric precision in the reconstruction [25].

**Boundary Overlap (BO):** Measures how well the edges or contours of a reconstructed model match those from the ground truth segmentation. It focuses on the accuracy of structural outlines, which is especially important for capturing fine details in plant morphology. BO is calculated as:

(6) $$\mathrm{BO}=\frac{|E_{\pi }\cap E_{\gamma }|}{|E_{\pi }\cup E_{\gamma }|}$$

here, $E_{\pi }$ denotes the set of edge pixels in the prediction, $E_{\gamma }$ refers to edge pixels from the ground truth, ∩ is the intersection, and ∪ is the union operators respectively. A higher BO value indicates better boundary agreement, reflecting sharper and more accurate structure definition [25,77].

**Precision, Recall, and F1-score:** These evaluation metrics are commonly used to assess the effectiveness of segmentation and detection in 3D plant modeling. They help determine how accurately relevant structures are identified and how thoroughly they are captured. Precision measures the proportion of correctly identified elements among all predicted positives:

(7) $$\mathrm{Precision}=\frac{TP}{TP+FP}$$

Recall evaluates the ability to capture all relevant elements from the ground truth:

(8) $$\mathrm{Recall}=\frac{TP}{TP+FN}$$

The F1-Score combines both metrics into a single value that reflects the balance between precision and recall:

(9) $$F1\text{-}\mathrm{score}=\frac{2\cdot (\mathrm{Precision}\times \mathrm{Recall})}{\mathrm{Precision}+\mathrm{Recall}}$$

here, TP stands for true positives, FP for false positives, and FN for false negatives. High values across these metrics indicate effective and reliable segmentation performance [76,77].

**Accuracy:** The 2D error distance when projecting reconstructed points back onto the image plane. It quantifies how closely the reconstruction matches the ground-truth projection. Lower accuracy error indicates more precise geometry and camera alignment [78].

**Completeness:** Completeness measures how fully the reconstructed 3D structure captures the ground-truth geometry. It quantifies missing or unreconstructed regions in the scene. Higher completeness means more of the plant structure is accurately captured [78].

**Matching Accuracy:** Matching accuracy reflects how well 2D image features align with reconstructed 3D points. During triangulation, consistent 2D–3D correspondences generate more stable 3D points. A higher number of reliable 3D points, therefore, indicates stronger matching accuracy [78].

#### 3.4.3. Trait-Specific Level Evaluation Metrics

Trait-level metrics quantitatively assess the accuracy of predicted plant features such as height, biomass, and leaf area from reconstructed models. These metrics are essential for validating the reliability of 3D data in phenotypic analysis.

**Coefficient of determination ($R^{2}$):** The $R^{2}$ score measures how well a reconstructed model captures the variability of plant trait values by comparing predicted outputs to actual observations:

(10) $$R^{2}=1-\frac{\sum_{j=1}^{M}{\left(\phi_{j}-{\hat{\phi }}_{j}\right)}^{2}}{\sum_{j=1}^{M}{\left(\phi_{j}-{\bar{\phi }}_{j}\right)}^{2}}$$

where $\phi_{j}$ the ground truth trait values (e.g., height or leaf area), ${\hat{\phi }}_{j}$ are the predicted values, and ${\bar{\phi }}_{j}$ is their means. A value of $R^{2}$ close to 1 indicates that the reconstruction effectively explains trait variation, supporting its use in phenotypic assessments [77].

**Root Mean Squared Error (RMSE):** RMSE measures the average magnitude of the errors between predicted and observed trait values by calculating the square root of the mean squared differences:

(11) $$\mathrm{RMSE}=\sqrt{\frac{1}{M}\sum_{j=1}^{M}{\left(\phi_{j}-{\hat{\phi }}_{j}\right)}^{2}}$$

here, $\phi_{j}$ represents the actual measurements, and ${\hat{\phi }}_{j}$ denotes the corresponding predictions. Lower RMSE values reflect higher accuracy, making this metric particularly important for evaluating biomass estimates in phenotypic studies [25,77].

**Mean Absolute Percentage Error (MAPE):** MAPE evaluates the average relative error between predicted and actual trait values, expressed as a percentage:

(12) $$\mathrm{MAPE}=\frac{1}{M}\sum_{j=1}^{M}\left|\frac{\psi_{j}-{\hat{\psi }}_{j}}{\psi_{j}}\right|\times 100\%$$

where $\psi_{j}$ represents the observed trait values and ${\hat{\psi }}_{j}$ is the corresponding predictions. A lower MAPE indicates greater accuracy in the reconstructed trait estimations, which is critical for reliable phenotypic analysis [9,25].

Table 3 provides an overview of these metrics, detailing their evaluation principles, ideal results, and specific significance in the context of plant phenotyping.

Despite the availability of diverse evaluation metrics, their adoption in plant phenotyping studies remains inconsistent. As observed in Table 4, most crop-specific studies report only a subset of metrics, primarily $R^{2}$ and RMSE for trait estimation, whereas pixel-level metrics such as SSIM and PSNR are predominantly used in neural rendering approaches. A comparative analysis indicates that NeRF-based methods typically achieve higher SSIM values (often >0.90) owing to superior texture reconstruction, whereas SfM–MVS pipelines demonstrate stronger geometric consistency, as reflected in lower Chamfer Distance and higher completeness. However, direct comparisons across studies remain challenging because of differences in datasets, sensor configurations, and evaluation protocols. This highlights the need for standardized benchmarking frameworks that integrate pixel-level, geometric, and trait-specific metrics into a unified evaluation pipeline. This heterogeneity highlights the need for future studies to report at least one geometry-level metric and one trait-level metric alongside any rendering-based measure when the intended application is plant phenotyping, rather than image synthesis alone.

### 3.5. Crop-Wise 3D Plant Reconstruction for Phenotype Analysis

Crop phenotyping involves assessing a wide array of complex traits related to plant growth, productivity, and responses to environmental stress, spanning scales from individual plant organs to full canopies [79]. These phenotypic traits include characteristics such as root architecture, biomass accumulation, leaf morphology, fruit properties, yield determinants, photosynthetic performance, and tolerance to abiotic stress [17]. The integration of 3D reconstruction techniques has advanced the field by enabling rapid, automated, and non-invasive phenotyping processes that can be repeated over time to monitor crop development dynamically [80]. This approach plays a pivotal role in capturing detailed structural traits, analyzing plant architecture, and linking physical form with functional traits [81]. In recent developments, a growing number of studies have explored the use of diverse sensor technologies to enhance the precision and throughput of phenotyping through 3D reconstruction methods. A comprehensive overview of such applications based on the crop types is presented in Table 4.

**Maize:** Several advanced 3D reconstruction methods have been applied to maize phenotyping, enabling precise, high-throughput analysis of structural traits critical for plant growth assessment. For example, Zhu et al. [82] utilized a line laser scanner integrated into the LemnaTec system, applying SfM, surface fitting, and edge detection techniques to reconstruct detailed leaf structures, supporting parameter space exploration. To capture morphological traits like leaf area and inclination angle, Thapa et al. [83] developed a LiDAR-based system using time-of-flight (ToF) measurements, enhancing non-destructive leaf trait quantification. Wu et al. [84] introduced a cost-effective, portable stereo vision-based platform (MVS-Pheno), employing MVS to measure traits such as plant height, leaf width, and leaf area in maize shoots. Further refining semantic analysis, Wen et al. [85] implemented triangular meshing and semantic point extraction using a 3D scan arm for accurate reconstruction of maize leaves. Li et al. [86] leveraged multi-view imaging with the FSFE-3200D-10GE system to extract comprehensive seedling phenotypes. Lastly, Guan et al. [87] applied an enhanced RANSAC-TrICP algorithm and DBSCAN clustering on Kinect v2 sensor data to recognize and assess maize stem and leaf traits across developmental stages.

**Lettuce:** Lettuce, a widely cultivated leafy vegetable in both field and controlled environments, requires precise phenotypic monitoring to enhance productivity and support breeding efforts. To support these efforts, Hu et al. [88] utilized a Kinect v2 camera and triangulation to automatically measure key growth parameters of lettuce, facilitating real-time, non-invasive growth assessment. To estimate structural traits such as volume and fresh weight, Bloch et al. [89] applied Intel RealSense RGB-D cameras and SDK-based point cloud processing. Ma et al. [90] further advanced dynamic growth monitoring by integrating MVS reconstruction into a rail-driven high-throughput phenotyping platform, allowing time-series analysis of lettuce development in greenhouses. Building on this, Ge et al. [91] developed LettuceP3D, a tool based on the MVS-PhenoV2 system and SoftGroup algorithm, enabling detailed 3D phenotypic analysis of individual plants.

**Sorghum:** Recent developments in 3D reconstruction technologies have significantly advanced sorghum phenotyping in open-field conditions. Bao et al. [92] developed a field-based robotic system using twelve Point Grey stereo cameras to capture 3D structural traits of sorghum architecture through stereo vision, enabling detailed morphological assessment. Expanding on stereo vision methods, Xiang et al. [93] introduced PhenoStereo, a high-throughput system that employs Phoenix 3.2MP cameras, triangulation, and stereo matching algorithms to estimate stem diameter under field conditions. To achieve broader phenotypic coverage, James et al. [94] proposed a scalable UAV-based pipeline incorporating NeRF, which enabled the extraction of complex traits such as plant and panicle count, leaf angle, density, morphology, and canopy height from high-resolution point clouds.

**Wheat:** Technological advances in 3D reconstruction have enabled high-resolution, non-destructive phenotypic analysis of wheat across various growth stages and scales. Gu et al. [95] introduced MVS-Pheno V2, which uses KD-tree algorithms for phenotypic trait extraction by integrating point cloud data and virtual design optimization. Duan et al. [96] used a Canon PowerShot ELPH 110 HS camera combined with MVS-SfM techniques to dynamically quantify canopy structure for assessing early plant vigor. Liu et al. [78] developed RepC-MVSNet, a self-supervised SfM and MVS-based framework using a Raspberry Pi video monitoring system for detailed 3D reconstruction. High-fidelity structural modeling was demonstrated by Stuart et al. [65], who applied 3DGS and NeRF using RGB-D and Einstar scanners for accurate wheat plant reconstruction. Expanding to in-field applications, Zhang et al. [97] introduced Wheat3DGS, which leverages 3DGS and the Segment Anything Model (SAM) for 3D instance segmentation and head phenotyping using data from ETH Zurich’s Field Phenotyping Platform. At the kernel level, Wu et al. [98] developed a novel platform combining NIR (near-infrared) detection and omni-directional 3D reconstruction to analyze single wheat seed morphology.

**Tomato:** Advanced 3D reconstruction techniques have been extensively applied to capture detailed morphological traits of tomato plants at multiple scales. For example, Choi et al. [9] utilized a 6-DoF robot equipped with an IDS U3-36L0XC machine vision camera, employing NeRF for comprehensive morphological analysis. Rose et al. [99] applied a Pix4D Mapper combined with SFM-MVS to achieve accurate organ-level phenotyping using Canon EOS 450D imagery. Nguyen et al. [38] implemented stereo vision and structured light techniques with Canon EOS Rebel T3 and NIKKOR-P lenses to measure plant height, total leaf area, and shading area. Zheng et al. [63] advanced the real-time simulation of tomato models through a phone camera with LiDAR, integrating Tomato-NeRF and Statistical Outlier Removal (SOR) for enhanced reconstruction fidelity. Furthermore, Usenko et al. [100] combined LiCOR 3100C leaf scanning and Canon PowerShot SX70 HS imaging with point cloud processing algorithms such as Alpha Shape, Marching Cubes, Poisson surface reconstruction, and Ball Pivoting to estimate total leaf area in dwarf tomato plants.

**Strawberry:** Various 3D reconstruction technologies have been employed to capture key morphological and growth traits of strawberry plants. He et al. [101] utilized a Canon EOS 1200D camera combined with Agisoft Photoscan and MVS techniques to measure parameters including height, length, width, volume, calyx size, color, and achene count. Huang et al. [102] applied an iPhone 16 Pro Max and Planar-based Gaussian Splatting Reconstruction (PGSR) for detailed canopy volume estimation. Further, Li et al. [103] proposed a method integrating the Revopoint POP3 3D scanner with SoftGroup algorithms to detect and measure leaf area. Additionally, Saha et al. [104] employed a mobile LiDAR laser scanner (LMS511 pro) to monitor vegetative growth dynamically. These integrated methodologies demonstrate the efficacy of combining diverse imaging and sensing technologies with advanced computational frameworks to enable comprehensive and high-throughput phenotyping of strawberry crops.

**Corn:** Advanced 3D reconstruction methods have been employed to capture detailed morphological and growth traits of corn plants. Li and Tang [105] developed a low-cost system using the PMD Camboard nano with multi-view vision and TOF techniques for comprehensive morphological trait characterization. Lati et al. [106] utilized an RGB-D camera combined with stereo vision to estimate growth parameters from sparse 3D reconstructions based on highly textured feature points. To overcome occlusion challenges, Gao et al. [107] applied a ZED2i stereo depth camera integrated with Shape Coding PointAttN (SCPAN) and multilayer perceptron models to reconstruct the complete shape and pose of corn plants. Additionally, Wei et al. [108] used a consumer-grade L515 LiDAR sensor with an automatic viewpoint planning approach to enable fast multi-view 3D reconstruction of seedlings.

**Cotton:** Recent advances in 3D reconstruction have facilitated high-throughput, precise phenotyping of cotton plants using diverse sensing technologies and algorithms. For example, Xiao et al. [109] employed UAV-based SfM-MVS technology to capture and characterize cotton bolls in situ at an organ scale. Li et al. [110] used RGB-D sensors combined with Instance Segmentation Networks (ISNs), Generative Adversarial Networks (GANs), and Point-cloud Reconstruction Algorithms (PRAs) to reconstruct complex canopy structures, including occluded internal regions. Jiang et al. [111] integrated smartphone and LiDAR sensors with SAM, YOLOv11x, and 3DGS to map cotton bolls and analyze plant architectural traits accurately. Hao et al. [112] utilized RGB cameras paired with the MVS-PhenoV2 platform and PointNet++ MSG for automatic phenotype extraction and wilting assessment from point clouds. More recently, Chu et al. [113] leveraged smartphone-acquired data with a NeRF-based Luma AI model for comprehensive 3D phenotype extraction across multiple cotton organs throughout the growing season.

**Soybean:** Low-cost 3D reconstruction techniques using Canon 500D cameras combined with SfM-MVS and VisualSFM have been employed to quantify key traits such as leaf length, leaf width, plant height, and leaf area, as demonstrated by Zhu et al. [114]. Wang et al. [115] utilized Kinect sensors alongside RANSAC and iterative closest point (ICP) algorithms to reconstruct soybean canopy morphology and extract detailed plant traits. More recently, Xin et al. [116] advanced in-field 3D reconstruction by integrating smartphone-acquired images with SfM and Instant-NGP, enabling rapid and accurate modeling of soybean plant structures. Sun et al. [117] improved phenotypic data acquisition at the vegetative stage through an automated image preprocessing pipeline employing DeepLabv3+ semantic segmentation combined with MVS using Canon EOS600D cameras. Furthermore, Cui et al. [118] applied multi-view stereo 3D reconstruction paired with the Point Voxel Segmentation Network (PVSegNet) and high-resolution Sony A7 cameras for automated phenotypic analysis of mature soybean plants, facilitating precise trait quantification.

**Table 4:** Crop-wise summary of studies on 3D plant reconstruction for phenotypic measurement.

Crop	Authors	Application	Algorithm	Performance	GT	Limitation
Maize	Zhu et al. (2018) [82]	Parameter space exploration	SFM, Surface/ Edge fitting	Avg variances: 28.7%	NA	Not suitable for complex structures.
	Thapa et al. (2018) [83]	Leaf area/ inclination	TOF	$R^{2}>0.95$	LI-3100C	1. Simple crops only; 2. Manual params; 3. Stem removal issues.
	Wu et al. (2020) [84]	Height, width, area	MVS	$R^{2}$: 0.99, 0.87, 0.93	FARO scanner	Maize-specific; requires shoot fit; species-dependent.
	Wen et al. (2024) [85]	Semantic feature points	Triangular meshing	Error: 0.5–0.8 cm	MM	Needs high-quality points; not generalized; occlusion.
	Li et al. (2022) [86]	Phenotypic params	SfM	$R^{2}$: 0.99, 0.98, 0.92	Calipers	Time consuming.
	Guan et al. (2025) [87]	Growth profiling	RANSAC-TrICP, DBSCAN	$R^{2}>0.97$	MM	Growth state variability affects accuracy.
Lettuce	Hu et al. (2018) [88]	Growth measurement	Triangulation	$R^{2}>0.95$	MM	Stable lighting needed; specific pot settings.
	Bloch et al. (2025) [89]	Fresh weight	Vacuum package	RMSE 18.2 g	Scale	No internal structure capture; lacks generality.
	Ma et al. (2025) [90]	Dynamics monitoring	MVS	$R^{2}$: 0.79, 0.60	MM	Limited area; low point resolution.
	Ge et al. (2025) [90]	3D analysis	ContextCapture	mIoU: 86.7%	MM	Time-consuming; stable lighting required.
Sorghum	Bao et al. (2019) [92]	Field phenotyping	Stereo view	Stem $R^{2}=0.96$	MM	Computationally inefficient.
	Xiang et al. (2020) [93]	Stem diameter	Stereo matching	MAE: 1.44 mm	MM	Limited to stem diameter only.
	James et al. (2025) [94]	Panicle count/ morphology	NeRF	0.850 mAP	MM	Computationally heavy.
Wheat	Gu et al. (2024) [95]	Phenotypic extraction	KD-tree	$R^{2}$ height: 0.80	3D digitizer	Not scalable; 1:1 reconstruction difficult.
	Stuart et al. (2025) [65]	3D reconstruction	3DGS, NeRF	SSIM: 0.95	Einstar	Alignment and collision avoidance issues.
	Zhang et al. (2025) [97]	Instance Seg.	3DGS, SAM	PSNR: 25.447	FARO Focus	Failed at lower canopy/ wheat head levels.
Tomato	Choi et al. (2024) [9]	Morphological analysis	NeRF	Fruit $R^{2}:0.96$	MM	Manual region extraction limits scalability.
	Rose et al. (2015) [99]	Organ-level pheno	Pix4D, SFM-MVS	Leaf area $R^{2}=0.99$	Perceptron	Time consuming; limited automation.
	Zheng et al. (2024) [63]	Real-time simulation	Tomato-NeRF	PSNR: 27.55	Freescan	Resource intensive; complex data prep.
Cotton	Jiang et al. (2025) [111]	Boll mapping	SAM, YOLO, 3DGS	MAPE boll: 9.23%	FARO Focus	Needs precise lighting; complex algorithm.
	Hao et al. (2024) [112]	Extraction	PointNet++ MSG	Height $R^{2}:0.99$	NA	Not suitable for complex blade structures.
	Chu et al. (2025) [113]	Data extraction	Luma AI (NeRF)	mIoU: 67.55%	MM	Noisy point clouds in field environments.

**Synthesis: Method Suitability Across Crop Architectures and Deployment Settings**

The crop-wise analysis reveals that the performance of 3D reconstruction methods is highly dependent on plant architecture, sensing modality, and environmental conditions, rather than a single universally optimal approach. Previous studies have consistently shown that the structural complexity of plants significantly affects reconstruction accuracy, particularly in terms of occlusion, texture availability, and geometric continuity [68].

For crops with relatively smooth and continuous surfaces, such as lettuce and soybean, MVS and RGB-D-based approaches provide reliable reconstruction due to sufficient feature correspondences and moderate occlusion [5,55]. In contrast, graminaceous crops such as wheat, maize, and sorghum exhibit thin leaves, overlapping canopies, and high self-occlusion, which challenge image-based methods. In such cases, LiDAR-based systems have demonstrated improved robustness, as they are less sensitive to illumination and texture limitations [8,83].

Neural rendering approaches, including NeRF and 3DGS, have recently emerged as promising alternatives by learning continuous scene representations from multi-view images [12]. These methods achieve high visual fidelity and improved reconstruction under sparse viewpoints. However, their applicability in agricultural environments remains constrained. NeRF is computationally intensive and sensitive to large-scale scenes and dynamic conditions [25], while 3DGS, despite its efficiency, may introduce over-smoothing and geometric artifacts in thin plant structures due to its Gaussian representation [25].

From a deployment perspective, greenhouse environments favor RGB-D and multi-view imaging systems due to controlled lighting and stable acquisition conditions, enabling accurate and repeatable phenotyping [88,90]. In contrast, field environments require scalable solutions such as UAV-based imaging and LiDAR, which can handle large spatial coverage but often suffer from environmental variability, including wind, illumination changes, and occlusion [92].

Overall, these findings indicate that method selection should be guided by three key factors: (i) plant morphology, (ii) environmental conditions, and (iii) phenotyping objectives. Future research should therefore focus on hybrid frameworks that integrate the geometric robustness of classical methods with the flexibility of neural representations for improved generalization and scalability.

Based on the reviewed literature, several practical guidelines can be derived for selecting appropriate 3D reconstruction pipelines in plant phenotyping:

- **Leafy crops (e.g., lettuce, soybean)**: MVS and RGB-D systems are generally effective due to relatively smooth surfaces and lower occlusion. These methods provide reliable geometric reconstruction with moderate computational cost [5,55]. However, their performance may degrade in low-texture regions or under non-uniform illumination.
- **Graminaceous crops (e.g., wheat, maize, sorghum)**: LiDAR-based and hybrid reconstruction approaches are preferred due to their robustness to thin structures and dense canopy arrangements. Image-based methods alone often struggle with self-occlusion and feature sparsity [8,83].
- **Fruit-bearing crops (e.g., tomato, strawberry, cotton)**: Neural rendering methods such as NeRF and 3DGS have shown strong performance in capturing detailed morphological traits and fruit structures, particularly in controlled environments [9,12]. However, these approaches require dense multi-view data and significant computational resources, limiting their scalability.
- **Greenhouse environments**: RGB-D cameras, structured light systems, and multi-view imaging are well suited due to stable lighting conditions and controlled acquisition setups, enabling high-precision and repeatable phenotyping [88,90].
- **Field environments**: UAV-based imaging, LiDAR, and large-scale SfM–MVS pipelines are more practical for large-area coverage. However, reconstruction quality is often affected by illumination variability, wind-induced motion, and occlusion [92].
- **Trade-off consideration**: Classical methods prioritize geometric accuracy and scalability, whereas neural methods emphasize visual realism and flexibility. The optimal choice depends on whether the application focuses on quantitative trait extraction or high-quality visual reconstruction [4,68].

### 3.6. Some Publicly Available Software for 3D Plant Reconstruction

Several software solutions support image-based 3D reconstruction and photogrammetry, differing in their computational needs and capabilities (Table 5). A major distinction is distributed processing: open-source tools such as OpenDroneMap (using SfM and MVS) and Meshroom (SIFT, SfM, LSCM, ABF) support it, enabling large-scale mapping, whereas commercial solutions such as RealityCapture (SfM and multi-view geometry), Agisoft Metashape (SfM, MVS), 3DF Zephyr (SfM, ICP, Multi-ICP), Pix4DMapper (SfM, Bundle Block Adjustment, YOLO), and DJI Terra (3DGS, SfM, PPK) generally do not. Operating system support also varies among different devices. OpenDroneMap and Meshroom are cross-platform, whereas most commercial tools are limited to 64-bit Windows or Linux. Hardware demands range from 4 GB RAM (OpenDroneMap) to 32 GB or more (Agisoft, DJI Terra, 3DF Zephyr), with SSDs recommended for efficient data storage. GPU acceleration is central to this process. RealityCapture, Meshroom, and Metashape leverage CUDA-enabled GPUs for parallel computing, whereas 3DF Zephyr and Pix4DMapper rely more on CPUs with partial GPU use. DJI Terra integrates 3D Gaussian Splatting (3DGS) with SfM and requires high-performance NVIDIA GPUs. Cloud-based workflows remain limited, with OpenDroneMap among the few that support remote and distributed processing. Overall, open-source platforms provide flexible, research-oriented solutions. In contrast, commercial alternatives offer advanced pipelines, such as BBA in Pix4D, ICP refinements in 3DF Zephyr, or 3DGS in DJI Terra, optimized for professional applications. The selection of image-based software depends on several factors, including computational resources, the need for distributed processing, and specific project requirements. Understanding these differences is essential for choosing the most suitable software based on project scale, budget, and available computing infrastructure.

## 4. Challenges and Future Research Directions

### 4.1. Limitations of Current AI-Driven Plant Phenotyping Approaches

**Figure 6:**
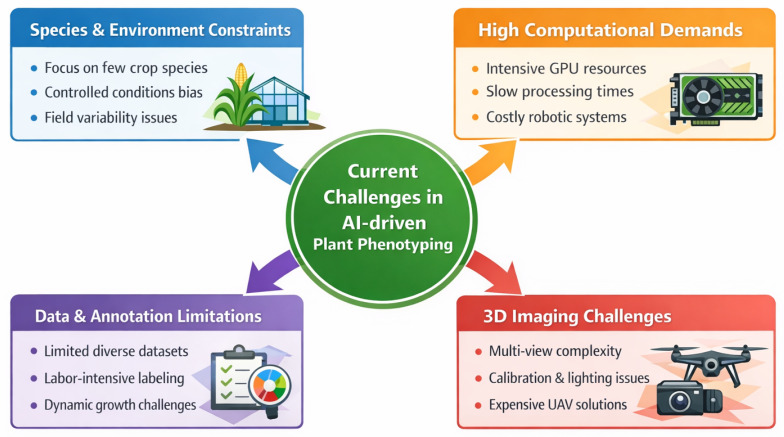
Limitations of the current 3D reconstruction approaches for plants.

Despite technological advancements, 3D reconstruction in agricultural scenarios continues to face significant challenges, as illustrated in Figure 6. These limitations arise not only from algorithmic constraints but also from practical issues related to scalability, acquisition complexity, and deployment feasibility of the data.

**Species and environmental constraints:** A critical limitation across many AI phenotyping studies is the restricted applicability of methods to specific crop species or controlled environments. Much of the recent work has predominantly focused on staple crops, such as maize and soybean [16,105], limiting generalization across diverse plant architectures. Structural variations in leaf morphology, canopy density, and growth patterns introduce inconsistencies in the reconstruction performance, particularly under field conditions. While greenhouse-based studies benefit from controlled illumination and minimal environmental disturbance, field environments introduce dynamic factors such as wind, illumination variability, and soil heterogeneity, which significantly degrade reconstruction accuracy [68,119]. These observations indicate that current methods are not yet robust enough for cross-species and cross-environment deployment.

**High computational demand (HCD):** Computational cost remains a primary bottleneck in scaling AI-driven phenotyping systems. Neural rendering approaches, such as NeRF, typically require several hours of GPU training (ranging from approximately 4 to 12 h per scene, depending on the resolution and number of views), making them impractical for high-throughput applications [120,121]. In comparison, 3D Gaussian Splatting (3DGS) significantly reduces the training time to the order of tens of minutes (approximately 30–60 min); however, this efficiency is achieved at the expense of reduced fidelity in complex plant structures [13,122]. Classical SfM–MVS pipelines, although less computationally demanding during inference, scale poorly with increasing image counts due to feature matching complexity. Furthermore, high-resolution image acquisition and precise sensor calibration impose additional computational and storage overheads, often exceeding the practical limits for real-time deployment [123,124]. These constraints highlight the trade-off between reconstruction quality, computational efficiency, and scalability.

**Data and annotation limitations:** The performance of AI models is inherently dependent on the availability of large-scale, diverse, and well-annotated datasets. Existing datasets, such as PlantGaussian [125] and Splants [126] provide valuable benchmarks but remain limited in terms of species diversity, environmental variation, and annotation richness. Moreover, annotation processes are labor-intensive, often requiring the manual labeling of complex plant structures, which significantly limits dataset scalability [127]. The lack of standardized evaluation protocols further complicates cross-study comparisons, resulting in inconsistent reporting of metrics such as $R^{2}$, RMSE, and SSIM [128]. These issues hinder reproducibility and limit the ability to establish unified benchmarks for the evaluation of models.

**3D imaging and acquisition challenges:** Multi-view imaging, which is fundamental to most reconstruction pipelines, presents significant logistical and operational challenges. High-quality reconstruction typically requires dense image capture (often exceeding 50–100 viewpoints per plant), which is difficult to achieve in large-scale field deployments [22]. In addition, factors such as camera calibration errors, motion blur, and inconsistent lighting introduce reconstruction artifacts and reduce geometric fidelity. Although UAV-based imaging improves scalability, it often sacrifices resolution and structural detail, particularly in lower canopy regions [129]. These limitations emphasize the need for efficient acquisition strategies that balance coverage, resolution, and operational feasibility of the data.

**Deployment and system complexity:** Beyond algorithmic limitations, the practical deployment of phenotyping systems remains constrained by cost, energy requirements, and system complexity. Although robotic platforms and multisensor setups are capable of high-throughput data collection, they are often expensive and require significant infrastructure, limiting their accessibility to large research facilities [130,131]. This creates a gap between laboratory-scale innovation and real-world agricultural adoption, particularly in resource-constrained environments.

### 4.2. Future Research Directions

**Figure 7:**
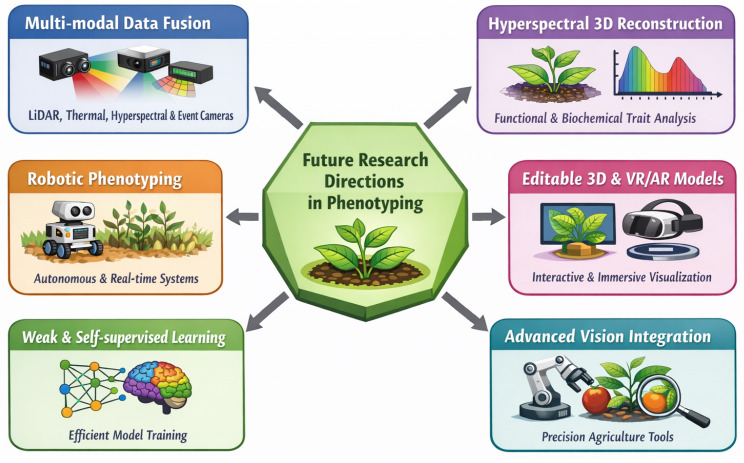
Future workflow landscape of the current 3D reconstruction for plant.

To address the limitations of current phenotyping approaches, future research must transition from exploratory development to structured, scalable, and standardized frameworks. This progression can be understood through a phased roadmap that distinguishes near-term priorities from longer-term research directions, as illustrated in Figure 7.

**Near-term priorities (practical and scalable advancements):**

*Benchmarking and metric standardization:* One of the most immediate needs is the development of standardized benchmarking protocols that unify pixel-level, geometric, and trait-specific evaluation metrics. Current studies report inconsistent metrics, limiting cross-method comparisons and reproducibility [16]. Establishing shared datasets and evaluation pipelines will enable more rigorous and comparable performance assessments.

*Lightweight and real-time deployment:* Reducing computational overhead is essential for practical adoption. Future efforts should focus on optimizing neural rendering pipelines, integrating model compression techniques, and leveraging edge computing to enable real-time or near-real-time phenotyping in field environments [121]. Lightweight architectures are critical for scaling laboratory prototypes to operational agricultural systems.

*Multi-modal data acquisition protocols:* Integrating complementary sensing modalities, including LiDAR, RGB-D, and thermal imaging, can improve reconstruction robustness under challenging environmental conditions [25,132]. Standardizing acquisition protocols, such as sensor placement, calibration procedures, and data synchronization, will further enhance consistency and model generalization.

**Longer-term priorities (emerging and transformative directions):**

*Editable plant digital twins:* The development of interactive and editable 3D plant models represents a significant step toward dynamic phenotyping systems. Techniques such as EditingNeRF and Gaussian-based editing frameworks enable the simulation of plant growth and environmental interactions, forming the foundation for digital twin systems [133,134,135].

*Cross-modal neural rendering:* Future reconstruction frameworks are expected to integrate spatial, spectral, and temporal information into unified representations. Cross-modal approaches that combine RGB, hyperspectral, and LiDAR data can improve both structural and functional phenotyping capabilities [136,137].

*Hyperspectral 3D phenotyping:* Incorporating hyperspectral imaging into 3D reconstruction pipelines enables simultaneous analysis of morphological and physiological traits, such as nutrient status and stress responses [26]. However, achieving real-time, high-resolution hyperspectral 3D reconstruction remains a challenge owing to computational and hardware constraints.

*AI-driven automation and downstream integration:* Integration with advanced computer vision models, including segmentation and detection frameworks, will enable automated trait extraction, disease monitoring, and yield prediction [68,138]. These developments are essential for translating reconstruction outputs into actionable agricultural insights.

**Overcoming Data Scarcity with Weak and Self-supervised Learning:** Given the laborious nature of data annotation, weak supervision and self-supervised learning techniques offer promising solutions to reduce dependency on large, annotated datasets [127]. By leveraging unlabeled or sparsely labeled data, these methods can improve model generalizability and training efficiency, particularly for complex phenotyping tasks involving irregular plant growth. Combining these learning paradigms with large-scale datasets, such as ShapeSplat [139] designed for self-supervised pretraining in 3D object representation, could accelerate progress in plant phenotyping applications.

## 5. Conclusions

This review presents a comprehensive synthesis of classical and deep learning-based 3D reconstruction methods, their underlying sensing systems, and their deployment in crop-specific applications. This study also demonstrates that 3D reconstruction methods for plant phenotyping exhibit distinct trade-offs rather than a single dominant solution. Classical approaches such as SfM–MVS and LiDAR provide robust geometric accuracy and are well suited for structured environments and metric trait extraction. In contrast, neural rendering approaches such as NeRF and 3DGS offer superior visual realism and improved reconstruction under sparse or unstructured data conditions, but remain constrained by computational cost and sensitivity to plant-specific challenges such as occlusion and fine-scale geometry. Additionally, evaluation metrics across pixel, geometry, and trait levels were discussed to support standardized benchmarking. Despite the notable progress, several limitations were identified. Most current 3D reconstruction systems face challenges in operating reliably under outdoor field conditions owing to variable lighting, occlusion, and environmental noise. Deep learning models, such as NeRF and 3DGS, while highly accurate, are often computationally intensive, limiting their real-time deployment on low-resource platforms. Furthermore, there is a lack of standard datasets, unified evaluation protocols, and generalizable frameworks that can be adapted to various crops, growth stages, and field conditions. Future research should prioritize the development of lightweight neural architectures, real-time model optimization, and hybrid sensor fusion systems to enhance robustness in dynamic agricultural environments. Ultimately, translating high-fidelity 3D reconstructions into practical, cost-effective field tools will be key to unlocking their full potential in data-driven agriculture.

## Figures and Tables

**Table 1:** Comparison of existing review papers on 3D reconstruction in agriculture.

Reviews	Traditional		DL-Based	Sensors/Systems	Algo. Synthesis	Evaluation Metrics	Crop-Specific
	Active	Passive					
Wei Guo et al. (2021) [20]	✓	✓	✗	✗	✗	✗	✗
Rijad Sarić et al. (2022) [26]	–	–	✗	✗	✗	✗	✗
S. Kolhar and J. Jagtap (2023) [21]	✓	✓	✗	✗	✗	✗	✗
M. Akhtar et al. (2024) [22]	✓	✓	✓	✗	✗	✗	✗
Yu et al. (2024) [24]	✓	✓	✗	✓	✗	✗	✗
Song et al. (2025) [23]	✓	✓	✗	✗	✗	✗	✗
Li et al. (2025) [25]	✓	✓	✓	✗	✗	✓	✗
Ours	✓	✓	✓	✓	✓	✓	✓

**Table 2:** State-of-the-art algorithms for 3D reconstruction.

Algorithm	Application	Input Data	Strengths	Weaknesses
Structure from Motion (SfM) [68]	3D reconstruction from 2D images	Multiple overlapping 2D images	High accuracy in point cloud generation; cost-effective	Sensitive to image quality and illumination variations
Multi-View Stereo (MVS) [67]	Dense 3D reconstruction from multiple views	Multiple calibrated 2D images from different perspectives	Produces detailed point clouds	Computationally intensive; requires numerous images
Time-of-Flight (ToF) Cameras [32]	Real-time 3D scanning	Depth data (point clouds)	Fast data acquisition; suitable for dynamic scenes	Limited resolution; affected by ambient light
LiDAR (Light Detection and Ranging) [69]	High-precision 3D scanning	Point cloud data from laser scans	High accuracy; effective in various lighting conditions	Expensive equipment; less effective for small scale features
Stereo Vision [54]	3D reconstruction using image pairs	Image pairs from two cameras	Cost-effective; real-time capability	Depth estimation errors in occluded regions
Neural Radiance Fields (NeRF) [70]	Photorealistic 3D scene reconstruction	2D images with known camera poses	Captures fine details; handles complex lighting	Requires extensive training data; computationally demanding
Gaussian Splatting [66]	Efficient 3D scene representation	Image sets from multiple views	Fast rendering; handles complex geometries	May produce artifacts in fine details; less mature than NeRF
Voxel Carving [71]	Shape reconstruction by carving a voxel grid	Binary silhouettes from multiple views	Suitable for simple structures; straightforward implementation	Limited precision for complex geometries; memory intensive
Deep Learning-based Reconstruction (e.g., CNNs) [27]	Feature learning and shape prediction	Labeled image datasets	Generalizes well from training data; handles complex shapes	Requires large annotated datasets; computationally expensive
Marching Cubes [74]	Surface reconstruction from volumetric data	Volumetric grid data (e.g., voxel grids)	Produces smooth surfaces; widely used	Susceptible to noise in input data; may miss fine details
Point Cloud NN (e.g., PointNet) [72]	Learning from unordered point clouds	Raw 3D point cloud data	Handles irregular data structures; robust to input permutations	Challenging to train; requires large datasets
Hybrid Methods (SfM + MVS) [73]	Combining multiple techniques for robust 3D reconstruction	Multiple 2D images and dense stereo data	Improved accuracy and completeness, leverages strengths of both methods	Increased computational complexity; requires careful integration

**Table 3:** 3D reconstruction evaluation metrics with preferred value directions (↑ = higher, ↓ = lower, →1 = closer to 1).

Category	Metrics	How It Works	Phenotypic Metrics of Relevance
Pixel level	PSNR (↑)	Calculates a logarithmic ratio between the peak possible signal and the error between images, typically measured in decibels	Indicates how well fine-grained surface details and textures are preserved
	LPIPS (↓)	Uses deep NN features to evaluate perceptual similarity between images	Measures the visual realism of textures and surface characteristics
	SSIM (→1)	Assesses image similarity by comparing luminance, contrast, and structural patterns	Assesses image similarity by comparing luminance, contrast, and structural patterns
Geometric	Boundary Overlap (↑)	Computes how closely predicted object edges align with true boundaries	Critical for capturing intricate outlines and fine structural edges
	Recall (↑)	Measures the proportion of actual plant features that were correctly identified by the model	Assesses the completeness of detected plant parts
	Precision (↑)	Calculates the proportion of correctly predicted plant features out of all predicted features	Indicates how effectively false positives are minimized
	F1 Score (↑)	Combines precision and recall into a single score using their harmonic mean	Provides a balanced view of detection performance for phenotypic traits
	Chamfer Distance (↓)	Averages the closest-point distance between two point clouds, in both directions	Quantifies geometric similarity and spatial alignment of the reconstructed shape
	Accuracy (↑)	Compares reconstructed points against ground-truth projections in 2D	Smaller 2D distances reflect higher reconstruction precision. Higher accuracy ensures reliable trait measurements
	Completeness (↑)	Compares coverage of reconstructed points against the true 3D surface	Higher completeness preserves full plant morphology for trait extraction
	Matching Accuracy (↑)	Triangulation refines 3D structure by aligning new 2D images to existing 3D points	Higher matching accuracy results in more reliable and denser 3D plant reconstruction
Trait-specific	$R^{2}$ Score (→1)	Shows how much of the variation in actual traits is captured by the prediction model	Reflects accuracy in estimating phenotypic measurements like height or leaf size
	MAPE (↓)	Averages the absolute percentage errors across predicted trait values	Indicates how well the model predicts quantitative phenotypic attributes
	RMSE (↓)	Computes the square root of the average squared difference between predicted and actual values	Useful for evaluating the closeness of pixel-level reconstructions

**Table 5:** Summary of software for 3D plant reconstruction. ^†^ denotes minimum and * denotes recommended hardware resources.

Software Name	Hardware Requirements	Free Acces	API	Distributed Processing	Web Address	Input Data Type	Alg. Used
OpenDroneMap	64-bit CPU ^†^; 20 GB disk ^†^; 4 GB RAM ^†^; latest CPU *; 100 GB disk *; 16 GB RAM *	Yes	Yes	Yes	https://www.opendronemap.org/	Aerial 2D images	SfM, MVS
RealityCapture	64-bit CPU ^†^; 8 GB RAM ^†^; NVIDIA CUDA 3.0+ GPU; 1 GB GPU RAM ^†^	No	Yes	N/A	https://www.capturingreality.com	Multiple 2D images	SfM, MvG
Meshroom	Intel i7/Ryzen 7 *; 8 GB RAM ^†^; CUDA GPU ^†^; 20 GB+ storage *; GTX 1070 *	Yes	N/A	No	https://alicevision.org/#meshroom	Multiple 2D images	SIFT, SfM, LSCM, ABF
Agisoft Metashape	Intel/AMD 4–8 cores; GPU (>700 cores); 16–32 GB RAM	No	Yes	Yes	https://www.agisoft.com	Multiple 2D images	SfM, MVS
DJI Terra	64-bit CPU ^†^; NVIDIA GPU ^†^; 32 GB RAM ^†^; GTX 2070+ *	No	Yes	No	https://enterprise.dji.com/es/dji-terra	Multiple 2D images	3DGS, SfM, PPK
3DF Zephyr	64-bit CPU 2.0 GHz ^†^; 16 GB RAM ^†^; 32 GB RAM *; 20 GB storage	No	Yes	N/A	https://www.3dflow.net/3df-zephyr-photogrammetry-s	Multiple 2D images	SfM, ICP, Multi-ICP
Pix4DMapper	4 GB RAM ^†^; 16 GB RAM *; 15 GB SSD	No	Yes	No	https://www.pix4d.com/	Multiple 2D images	SfM, BBA, YOLO

## Data Availability

No new data were created or analyzed in this study. Data sharing is not applicable to this article.

## References

[1] Fiorani F., Schurr U. (2013). Future scenarios for plant phenotyping. Annu. Rev. Plant Biol..

[2] Anshori M.F., Dirpan A., Sitaresmi T., Rossi R., Farid M., Hairmansis A., Purwoko B., Suwarno W.B., Nugraha Y. (2023). An overview of image-based phenotyping as an adaptive 4.0 technology for studying plant abiotic stress: A bibliometric and literature review. Heliyon.

[3] Okura F. (2022). 3D modeling and reconstruction of plants and trees: A cross-cutting review across computer graphics, vision, and plant phenotyping. Breed. Sci..

[4] Gao T., Zhu F., Paul P., Sandhu J., Doku H.A., Sun J., Pan Y., Staswick P., Walia H., Yu H. (2021). Novel 3D imaging systems for high-throughput phenotyping of plants. Remote Sens..

[5] Ubbens J.R., Stavness I. (2017). Deep plant phenomics: A deep learning platform for complex plant phenotyping tasks. Front. Plant Sci..

[6] Arshad M.A., Jubery T., Afful J., Jignasu A., Balu A., Ganapathysubramanian B., Sarkar S., Krishnamurthy A. (2024). Evaluating neural radiance fields for 3D plant geometry reconstruction in field conditions. Plant Phenomics.

[7] Schonberger J.L., Frahm J.M. (2016). Structure-from-motion revisited. Proceedings of the IEEE Conference on Computer Vision and Pattern Recognition (CVPR).

[8] Lin Y. (2015). LiDAR: An important tool for next-generation phenotyping technology of high potential for plant phenomics?. Comput. Electron. Agric..

[9] Choi H.B., Park J.K., Park S.H., Lee T.S. (2024). NeRF-based 3D reconstruction pipeline for acquisition and analysis of tomato crop morphology. Front. Plant Sci..

[10] Hu K., Ying W., Pan Y., Kang H., Chen C. (2024). High-fidelity 3D reconstruction of plants using Neural Radiance Fields. Comput. Electron. Agric..

[11] Wu S., Wen W., Gou W., Lu X., Zhang W., Zheng C., Xiang Z., Chen L., Guo X. (2022). A miniaturized phenotyping platform for individual plants using multi-view stereo 3D reconstruction. Front. Plant Sci..

[12] Mildenhall B., Srinivasan P.P., Tancik M., Barron J.T., Ramamoorthi R., Ng R. (2021). Nerf: Representing scenes as neural radiance fields for view synthesis. Commun. ACM.

[13] Chen G., Wang W. (2024). A survey on 3d gaussian splatting. arXiv.

[14] Chen H., Liu S., Wang C., Wang C., Gong K., Li Y., Lan Y. (2023). Point cloud completion of plant leaves under occlusion conditions based on deep learning. Plant Phenomics.

[15] Bömer J., Esser F., Marks E., Rosu R.A., Behnke S., Klingbeil L., Kuhlmann H., Stachniss C., Mahlein A.K., Paulus S. (2024). A 3D printed plant model for accurate and reliable 3D plant phenotyping. GigaScience.

[16] Harandi N., Vandenberghe B., Vankerschaver J., Depuydt S., Van Messem A. (2023). How to make sense of 3D representations for plant phenotyping: A compendium of processing and analysis techniques. Plant Methods.

[17] Li L., Zhang Q., Huang D. (2014). A review of imaging techniques for plant phenotyping. Sensors.

[18] Costa C., Schurr U., Loreto F., Menesatti P., Carpentier S. (2019). Plant phenotyping research trends, a science mapping approach. Front. Plant Sci..

[19] Jiang Y., Li C. (2020). Convolutional neural networks for image-based high-throughput plant phenotyping: A review. Plant Phenomics.

[20] Guo W., Carroll M.E., Singh A., Swetnam T.L., Merchant N., Sarkar S., Singh A.K., Ganapathysubramanian B. (2021). UAS-based plant phenotyping for research and breeding applications. Plant Phenomics.

[21] Kolhar S., Jagtap J. (2023). Plant trait estimation and classification studies in plant phenotyping using machine vision–A review. Inf. Process. Agric..

[22] Akhtar M.S., Zafar Z., Nawaz R., Fraz M.M. (2024). Unlocking plant secrets: A systematic review of 3D imaging in plant phenotyping techniques. Comput. Electron. Agric..

[23] Song H., Wen W., Wu S., Guo X. (2025). Comprehensive review on 3D point cloud segmentation in plants. Artif. Intell. Agric..

[24] Yu S., Liu X., Tan Q., Wang Z., Zhang B. (2024). Sensors, systems and algorithms of 3D reconstruction for smart agriculture and precision farming: A review. Comput. Electron. Agric..

[25] Li J., Qi X., Nabaei S.H., Liu M., Chen D., Sun Q., Zhang X., Yin X., Li Z. (2025). A survey on 3D reconstruction techniques in plant phenotyping: From classical methods to neural radiance fields (NeRF), 3D gaussian splatting (3DGS), and beyond. Plant Phenomics.

[26] Sarić R., Nguyen V.D., Burge T., Berkowitz O., Trtílek M., Whelan J., Lewsey M.G., Čustović E. (2022). Applications of hyperspectral imaging in plant phenotyping. Trends Plant Sci..

[27] Samavati T., Soryani M. (2023). Deep learning-based 3D reconstruction: A survey. Artif. Intell. Rev..

[28] Zhou L., Wu G., Zuo Y., Chen X., Hu H. (2024). A comprehensive review of vision-based 3D reconstruction methods. Sensors.

[29] Moonrinta J., Chaivivatrakul S., Dailey M.N., Ekpanyapong M. (2010). Fruit detection, tracking, and 3D reconstruction for crop mapping and yield estimation. 2010 11th International Conference on Control Automation Robotics & Vision.

[30] Wang T.L., Ao L., Zheng J., Sun Z.B. (2023). Reconstructing depth images for time-of-flight cameras based on second-order correlation functions. Photonics.

[31] Cui Y., Schuon S., Chan D., Thrun S., Theobalt C. (2010). 3D shape scanning with a time-of-flight camera. 2010 IEEE Computer Society Conference on Computer Vision and Pattern Recognition.

[32] He Y., Chen S. (2019). Recent advances in 3D data acquisition and processing by time-of-flight camera. IEEE Access.

[33] Shrestha S., Heide F., Heidrich W., Wetzstein G. (2016). Computational imaging with multi-camera time-of-flight systems. ACM Trans. Graph. (ToG).

[34] Biskup B., Scharr H., Schurr U., Rascher U. (2007). A stereo imaging system for measuring structural parameters of plant canopies. Plant Cell Environ..

[35] Liu J.F., Yan J.H., Lo S. (2013). A new insertion sequence for incremental Delaunay triangulation. Acta Mech. Sin..

[36] Su T., Wang W., Lv Z., Wu W., Li X. (2016). Rapid Delaunay triangulation for randomly distributed point cloud data using adaptive Hilbert curve. Comput. Graph..

[37] Massot-Campos M., Oliver-Codina G. (2015). Optical sensors and methods for underwater 3D reconstruction. Sensors.

[38] Nguyen T.T., Slaughter D.C., Max N., Maloof J.N., Sinha N. (2015). Structured light-based 3D reconstruction system for plants. Sensors.

[39] Pintore G., Mura C., Ganovelli F., Fuentes-Perez L., Pajarola R., Gobbetti E. (2020). State-of-the-art in automatic 3D reconstruction of structured indoor environments. Comput. Graph. Forum.

[40] Cheng J., Chung C.K.R., Lam E.Y., Fung K.S., Wang F., Leung W. (2008). Structured-light based sensing using a single fixed fringe grating: Fringe boundary detection and 3-D reconstruction. IEEE Trans. Electron. Packag. Manuf..

[41] Mirdehghan P., Wu M., Chen W., Lindell D.B., Kutulakos K.N. (2024). Turbosl: Dense accurate and fast 3d by neural inverse structured light. Proceedings of the IEEE/CVF Conference on Computer Vision and Pattern Recognition.

[42] Siudak M., Rokita P. (2014). A survey of passive 3D reconstruction methods on the basis of more than one image. Mach. Graph. Vis..

[43] El hazzat S., Saaidi A., Satori K. (2014). Multi-view passive 3D reconstruction: Comparison and evaluation of three techniques and a new method for 3D object reconstruction. 2014 International Conference on Next Generation Networks and Services (NGNS).

[44] Li Z., Shi Y., Wang C., Wang Y. (2008). Accurate calibration method for a structured light system. Opt. Eng..

[45] Yang L., Tang R., Chen K. (2017). Call, put and bidirectional option contracts in agricultural supply chains with sales effort. Appl. Math. Model..

[46] Bruno F., Bianco G., Muzzupappa M., Barone S., Razionale A.V. (2011). Experimentation of structured light and stereo vision for underwater 3D reconstruction. ISPRS J. Photogramm. Remote Sens..

[47] Banz C., Hesselbarth S., Flatt H., Blume H., Pirsch P. (2010). Real-time stereo vision system using semi-global matching disparity estimation: Architecture and FPGA-implementation. 2010 International Conference on Embedded Computer Systems: Architectures, Modeling and Simulation.

[48] Chang X., Zhou Z., Wang L., Shi Y., Zhao Q. (2011). Real-time accurate stereo matching using modified two-pass aggregation and winner-take-all guided dynamic programming. 2011 International Conference on 3D Imaging, Modeling, Processing, Visualization and Transmission.

[49] Furukawa Y., Hernández C. (2015). Multi-view stereo: A tutorial. Found. Trends Comput. Graph. Vis..

[50] Koutsoudis A., Vidmar B., Ioannakis G., Arnaoutoglou F., Pavlidis G., Chamzas C. (2014). Multi-image 3D reconstruction data evaluation. J. Cult. Herit..

[51] Qi S., Ning X., Yang G., Zhang L., Long P., Cai W., Li W. (2021). Review of multi-view 3D object recognition methods based on deep learning. Displays.

[52] Zhang W., Liu Z., Zhou L., Leung H., Chan A.B. (2017). Martial arts, dancing and sports dataset: A challenging stereo and multi-view dataset for 3d human pose estimation. Image Vis. Comput..

[53] Koutsoudis A., Ioannakis G., Arnaoutoglou F., Kiourt C., Chamzas C. (2020). 3D reconstruction challenges using structure-from-motion. Applying Innovative Technologies in Heritage Science.

[54] Hossein-Nejad Z., Agahi H., Mahmoodzadeh A. (2021). Image matching based on the adaptive redundant keypoint elimination method in the SIFT algorithm. Pattern Anal. Appl..

[55] Lou L., Liu Y., Han J., Doonan J.H. (2014). Accurate multi-view stereo 3D reconstruction for cost-effective plant phenotyping. Image Analysis and Recognition: 11th International Conference, ICIAR 2014, Vilamoura, Portugal, 22–24 October 2014, Proceedings, Part II.

[56] Smith M.W., Carrivick J.L., Quincey D.J. (2016). Structure from motion photogrammetry in physical geography. Prog. Phys. Geogr..

[57] Javadnejad F., Slocum R.K., Gillins D.T., Olsen M.J., Parrish C.E. (2021). Dense point cloud quality factor as proxy for accuracy assessment of image-based 3D reconstruction. J. Surv. Eng..

[58] Fu K., Peng J., He Q., Zhang H. (2021). Single image 3D object reconstruction based on deep learning: A review. Multimed. Tools Appl..

[59] Chang A.X., Funkhouser T., Guibas L., Hanrahan P., Huang Q., Li Z., Savarese S., Savva M., Song S., Su H. (2015). Shapenet: An information-rich 3d model repository. arXiv.

[60] Xiang Y., Kim W., Chen W., Ji J., Choy C., Su H., Mottaghi R., Guibas L., Savarese S. (2016). Objectnet3d: A large scale database for 3d object recognition. Computer Vision—ECCV 2016: 14th European Conference, Amsterdam, The Netherlands, 11–14 October 2016, Proceedings, Part VIII.

[61] Xiang Y., Mottaghi R., Savarese S. (2014). Beyond pascal: A benchmark for 3d object detection in the wild. IEEE Winter Conference on Applications of Computer Vision.

[62] Geiger A., Lenz P., Urtasun R. (2012). Are we ready for autonomous driving? The kitti vision benchmark suite. 2012 IEEE Conference on Computer Vision and Pattern Recognition.

[63] Zheng X., Ai X., Qin H., Rong J., Zhang Z., Yang Y., Yuan T., Li W. (2024). Tomato-nerf: Advancing tomato model reconstruction with improved neural radiance fields. IEEE Access.

[64] Cheng K., Long X., Yang K., Yao Y., Yin W., Ma Y., Wang W., Chen X. (2024). Gaussianpro: 3d gaussian splatting with progressive propagation. Proceedings of the Forty-First International Conference on Machine Learning.

[65] Stuart L.A., Wells D.M., Atkinson J.A., Castle-Green S., Walker J., Pound M.P. (2025). High-fidelity wheat plant reconstruction using 3D Gaussian splatting and neural radiance fields. GigaScience.

[66] Kerbl B., Meuleman A., Kopanas G., Wimmer M., Lanvin A., Drettakis G. (2024). A hierarchical 3d gaussian representation for real-time rendering of very large datasets. ACM Trans. Graph. (TOG).

[67] Zhang J., Li S., Luo Z., Fang T., Yao Y. (2023). Vis-mvsnet: Visibility-aware multi-view stereo network. Int. J. Comput. Vis..

[68] Paturkar A., Sen Gupta G., Bailey D. (2022). Plant trait measurement in 3D for growth monitoring. Plant Methods.

[69] Hadadi M., Saraeian M., Godbersen J., Jubery T.Z., Li Y., Attigala L., Balu A., Sarkar S., Schnable P.S., Krishnamurthy A. (2025). Procedural generation of 3D maize plant architecture from LiDAR data. Comput. Electron. Agric..

[70] Yao M., Huo Y., Ran Y., Tian Q., Wang R., Wang H. (2024). Neural radiance field-based visual rendering: A comprehensive review. arXiv.

[71] Feng J., Saadati M., Jubery T., Jignasu A., Balu A., Li Y., Attigala L., Schnable P.S., Sarkar S., Ganapathysubramanian B. (2023). 3D reconstruction of plants using probabilistic voxel carving. Comput. Electron. Agric..

[72] Kumar A., Ray N., Panda R., Patra P.K., Das M. (2024). A Deep3D: Comprehensive Approaches for 3D Object Analysis and Feature Extraction Using PointNet. 2024 1st International Conference on Cognitive, Green and Ubiquitous Computing (IC-CGU).

[73] Morita M.M., Carvajal D.A.L., Bagur I.L.G., Bilmes G.M. (2024). A combined approach of SFM-MVS photogrammetry and reflectance transformation imaging to enhance 3D reconstructions. J. Cult. Herit..

[74] Chin D.J.Y., Mohamed A.S.A., Shariff K.A., Ishikawa K. (2021). GPU-Accelerated Enhanced Marching Cubes 33 for Fast 3D Reconstruction of Large Bone Defect CT Images. Advances in Visual Informatics: 7th International Visual Informatics Conference, IVIC 2021, Kajang, Malaysia, 23–25 November 2021, Proceedings.

[75] Zhu X., Huang Z., Li B. (2024). Three-dimensional phenotyping pipeline of potted plants based on neural radiation fields and path segmentation. Plants.

[76] Shen Y., Zhou H., Yang X., Lu X., Guo Z., Jiang L., He Y., Cen H. (2025). Biomass phenotyping of oilseed rape through UAV multi-view oblique imaging with 3DGS and SAM model. Comput. Electron. Agric..

[77] Yang X., Lu X., Xie P., Guo Z., Fang H., Fu H., Hu X., Sun Z., Cen H. (2024). PanicleNeRF: Low-cost, high-precision in-field phenotyping of rice panicles with smartphone. Plant Phenomics.

[78] Liu H., Xin C., Lai M., He H., Wang Y., Wang M., Li J. (2023). RepC-MVSNet: A reparameterized self-supervised 3D reconstruction algorithm for wheat 3D reconstruction. Agronomy.

[79] Ullah H.S., Bais A. (2022). Evaluation of model generalization for growing plants using conditional learning. Artif. Intell. Agric..

[80] Yang W., Feng H., Zhang X., Zhang J., Doonan J.H., Batchelor W.D., Xiong L., Yan J. (2020). Crop phenomics and high-throughput phenotyping: Past decades, current challenges, and future perspectives. Mol. Plant.

[81] Sunil G., Koparan C., Ahmed M.R., Zhang Y., Howatt K., Sun X. (2022). A study on deep learning algorithm performance on weed and crop species identification under different image background. Artif. Intell. Agric..

[82] Zhu F., Thapa S., Gao T., Ge Y., Walia H., Yu H. (2018). 3D reconstruction of plant leaves for high-throughput phenotyping. 2018 IEEE International Conference on Big Data (Big Data).

[83] Thapa S., Zhu F., Walia H., Yu H., Ge Y. (2018). A novel LiDAR-based instrument for high-throughput, 3D measurement of morphological traits in maize and sorghum. Sensors.

[84] Wu S., Wen W., Wang Y., Fan J., Wang C., Gou W., Guo X. (2020). MVS-Pheno: A portable and low-cost phenotyping platform for maize shoots using multiview stereo 3D reconstruction. Plant Phenomics.

[85] Wen W., Wu S., Lu X., Liu X., Gu S., Guo X. (2024). Accurate and semantic 3D reconstruction of maize leaves. Comput. Electron. Agric..

[86] Li Y., Liu J., Zhang B., Wang Y., Yao J., Zhang X., Fan B., Li X., Hai Y., Fan X. (2022). Three-dimensional reconstruction and phenotype measurement of maize seedlings based on multi-view image sequences. Front. Plant Sci..

[87] Guan H., Zhang X., Ma X., Zhuo Z., Deng H. (2025). Recognition and phenotypic detection of maize stem and leaf at seedling stage based on 3D reconstruction technique. Opt. Laser Technol..

[88] Hu Y., Wang L., Xiang L., Wu Q., Jiang H. (2018). Automatic non-destructive growth measurement of leafy vegetables based on kinect. Sensors.

[89] Bloch V., Shapiguzov A., Kotilainen T., Pastell M. (2025). A method for phenotyping lettuce volume and structure from 3D images. Plant Methods.

[90] Ma H., Wen W., Gou W., Lu X., Fan J., Zhang M., Liang Y., Gu S., Guo X. (2025). 3D time-series phenotyping of lettuce in greenhouses. Biosyst. Eng..

[91] Ge X., Wu S., Wen W., Shen F., Xiao P., Lu X., Liu H., Zhang M., Guo X. (2025). LettuceP3D: A tool for analysing 3D phenotypes of individual lettuce plants. Biosyst. Eng..

[92] Bao Y., Tang L., Breitzman M.W., Salas Fernandez M.G., Schnable P.S. (2019). Field-based robotic phenotyping of sorghum plant architecture using stereo vision. J. Field Robot..

[93] Xiang L., Tang L., Gai J., Wang L. (2020). PhenoStereo: A high-throughput stereo vision system for field-based plant phenotyping-with an application in sorghum stem diameter estimation. Proceedings of the 2020 ASABE Annual International Virtual Meeting.

[94] James C., Chandra S.S., Chapman S.C. (2025). A scalable and efficient UAV-based pipeline and deep learning framework for phenotyping sorghum panicle morphology from point clouds. Plant Phenomics.

[95] Gu W., Wen W., Wu S., Zheng C., Lu X., Chang W., Xiao P., Guo X. (2024). 3D reconstruction of wheat plants by integrating point cloud data and virtual design optimization. Agriculture.

[96] Duan T., Chapman S., Holland E., Rebetzke G., Guo Y., Zheng B. (2016). Dynamic quantification of canopy structure to characterize early plant vigour in wheat genotypes. J. Exp. Bot..

[97] Zhang D., Gajardo J., Medic T., Katircioglu I., Boss M., Kirchgessner N., Walter A., Roth L. (2025). Wheat3dgs: In-field 3d reconstruction, instance segmentation and phenotyping of wheat heads with gaussian splatting. Proceedings of the IEEE/CVF Conference on Computer Vision and Pattern Recognition (CVPR) Workshops.

[98] Wu T., Dai J., Shen P., Liu H., Wei Y. (2023). Seedscreener: A novel integrated wheat germplasm phenotyping platform based on NIR-feature detection and 3D-reconstruction. Comput. Electron. Agric..

[99] Rose J.C., Paulus S., Kuhlmann H. (2015). Accuracy analysis of a multi-view stereo approach for phenotyping of tomato plants at the organ level. Sensors.

[100] Usenko D., Helman D., Giladi C. (2025). Using 3D reconstruction from image motion to predict total leaf area in dwarf tomato plants. Comput. Electron. Agric..

[101] He J.Q., Harrison R.J., Li B. (2017). A novel 3D imaging system for strawberry phenotyping. Plant Methods.

[102] Huang Z., Lee W.S., Zhang P., Jeon H., Zhu H. (2025). SASP: Segment any strawberry plant, an end-to-end strawberry canopy volume estimation. Smart Agric. Technol..

[103] Li Z., Wang S., Su Y., Yu D. (2025). A method for measuring strawberry leaf area based on three-dimensional point cloud instance segmentation. IEEE Access.

[104] Saha K.K., Tsoulias N., Weltzien C., Zude-Sasse M. (2022). Estimation of vegetative growth in strawberry plants using mobile LiDAR laser scanner. Horticulturae.

[105] Li J., Tang L. (2017). Developing a low-cost 3D plant morphological traits characterization system. Comput. Electron. Agric..

[106] Lati R.N., Filin S., Eizenberg H. (2013). Plant growth parameter estimation from sparse 3D reconstruction based on highly-textured feature points. Precis. Agric..

[107] Gao Y., Li Z., Liu T., Li B., Zhang L. (2025). Point Cloud Completion of Occluded Corn with a 3D Positional Gated Multilayer Perceptron and Prior Shape Encoder. Agronomy.

[108] Wei K., Liu S., Chen Q., Huang S., Zhong M., Zhang J., Sun H., Wu K., Fan S., Ye Z. (2024). Fast Multi-View 3D reconstruction of seedlings based on automatic viewpoint planning. Comput. Electron. Agric..

[109] Xiao S., Fei S., Ye Y., Xu D., Xie Z., Bi K., Guo Y., Li B., Zhang R., Ma Y. (2024). 3D reconstruction and characterization of cotton bolls in situ based on UAV technology. ISPRS J. Photogramm. Remote Sens..

[110] Li Y., Si S., Liu X., Zou L., Wu W., Liu X., Zhang L. (2023). Three-dimensional reconstruction of cotton plant with internal canopy occluded structure recovery. Comput. Electron. Agric..

[111] Jiang L., Sun J., Chee P.W., Li C., Fu L. (2025). Cotton3DGaussians: Multiview 3D Gaussian Splatting for boll mapping and plant architecture analysis. Comput. Electron. Agric..

[112] Hao H., Wu S., Li Y., Wen W., Zhang Y., Zhuang L., Xu L., Li H., Guo X., Liu S. (2024). Automatic acquisition, analysis and wilting measurement of cotton 3D phenotype based on point cloud. Biosyst. Eng..

[113] Chu P., Han B., Guo Q., Wan Y., Zhang J. (2025). A three-dimensional phenotype extraction method based on point cloud segmentation for all-period cotton multiple organs. Plants.

[114] Zhu R., Sun K., Yan Z., Yan X., Yu J., Shi J., Hu Z., Jiang H., Xin D., Zhang Z. (2020). Analysing the phenotype development of soybean plants using low-cost 3D reconstruction. Sci. Rep..

[115] Wang F., Ma X., Liu M., Wei B. (2022). Three-dimensional reconstruction of soybean canopy based on multivision technology for calculation of phenotypic traits. Agronomy.

[116] Cao X., Qin X., Xu X. (2025). Three-demensional reconstruction of soybean plants in the field based on SfM and Instant-NGP. Trans. Chin. Soc. Agric. Eng..

[117] Sun Y., Miao L., Zhao Z., Pan T., Wang X., Guo Y., Xin D., Chen Q., Zhu R. (2023). An efficient and automated image preprocessing using semantic segmentation for improving the 3D reconstruction of soybean plants at the vegetative stage. Agronomy.

[118] Cui D., Liu P., Liu Y., Zhao Z., Feng J. (2025). Automated phenotypic analysis of mature soybean using multi-view stereo 3D reconstruction and point cloud segmentation. Agriculture.

[119] Paturkar A., Gupta G.S., Bailey D. (2020). Non-destructive and cost-effective 3D plant growth monitoring system in outdoor conditions. Multimed. Tools Appl..

[120] Pongpiyapaiboon S., Tanaka H., Hashiguchi M., Hashiguchi T., Hayashi A., Tanabata T., Isobe S., Akashi R. (2023). Development of a digital phenotyping system using 3D model reconstruction for zoysiagrass. Plant Phenome J..

[121] Zhao J., Ying W., Pan Y., Yi Z., Chen C., Hu K., Kang H. (2024). Exploring accurate 3d phenotyping in greenhouse through neural radiance fields. arXiv.

[122] Qin M., Li W., Zhou J., Wang H., Pfister H. (2024). Langsplat: 3d language gaussian splatting. Proceedings of the IEEE/CVF Conference on Computer Vision and Pattern Recognition.

[123] Das Choudhury S., Maturu S., Samal A., Stoerger V., Awada T. (2020). Leveraging image analysis to compute 3D plant phenotypes based on voxel-grid plant reconstruction. Front. Plant Sci..

[124] Uchida M., Okumura H., Tanaka M., Fukuda O., Yamaguchi N., Yeoh W.L. (2024). Monitoring of plant leaf growth based on 3D point cloud data. Remote Sensing for Agriculture, Ecosystems, and Hydrology XXVI.

[125] Shen P., Jing X., Deng W., Jia H., Wu T. (2025). PlantGaussian: Exploring 3D Gaussian splatting for cross-time, cross-scene, and realistic 3D plant visualization and beyond. Crop J..

[126] Ojo T., La T., Morton A., Stavness I. (2024). Splanting: 3d plant capture with gaussian splatting. SIGGRAPH Asia 2024 Technical Communications.

[127] Luo L., Jiang X., Yang Y., Samy E.R.A., Lefsrud M., Hoyos-Villegas V., Sun S. (2023). Eff-3dpseg: 3d organ-level plant shoot segmentation using annotation-efficient deep learning. Plant Phenomics.

[128] Phan T.T.H., Ngo T.M.V., Phan H.P. (2024). Flexible mechanical sensors for plant growth monitoring: An emerging area for smart agriculture. Sensors.

[129] Müller T., Evans A., Schied C., Keller A. (2022). Instant neural graphics primitives with a multiresolution hash encoding. ACM Trans. Graph. (TOG).

[130] Esser F., Rosu R.A., Cornelißen A., Klingbeil L., Kuhlmann H., Behnke S. (2023). Field robot for high-throughput and high-resolution 3d plant phenotyping: Towards efficient and sustainable crop production. IEEE Robot. Autom. Mag..

[131] Yao L., Van De Zedde R., Kowalchuk G. (2021). Recent developments and potential of robotics in plant eco-phenotyping. Emerg. Top. Life Sci..

[132] Zhang J., Zhang F., Kuang S., Zhang L. (2024). Nerf-lidar: Generating realistic lidar point clouds with neural radiance fields. Proc. Aaai Conf. Artif. Intell..

[133] Liu S., Zhang X., Zhang Z., Zhang R., Zhu J.Y., Russell B. (2021). Editing conditional radiance fields. Proceedings of the IEEE/CVF International Conference on Computer Vision.

[134] Ye M., Danelljan M., Yu F., Ke L. (2024). Gaussian grouping: Segment and edit anything in 3d scenes. Computer Vision—ECCV 2024: 18th European Conference, Milan, Italy, 29 September–4 October 2024, Proceedings, Part XXIX.

[135] Huang Y.H., Sun Y.T., Yang Z., Lyu X., Cao Y.P., Qi X. (2024). Sc-gs: Sparse-controlled gaussian splatting for editable dynamic scenes. Proceedings of the IEEE/CVF Conference on Computer Vision and Pattern Recognition.

[136] Chen G., Narayanan S.K., Ottou T.G., Missaoui B., Muriki H., Pradalier C., Chen Y. (2024). Hyperspectral neural radiance fields. arXiv.

[137] Zhang L., Pan T., Liu J., Han L. (2024). Compressing hyperspectral images into multilayer perceptrons using fast-time hyperspectral neural radiance fields. IEEE Geosci. Remote Sens. Lett..

[138] Kirillov A., Mintun E., Ravi N., Mao H., Rolland C., Gustafson L., Xiao T., Whitehead S., Berg A., Lo W. (2023). Segment anything. Proceedings of the IEEE/CVF International Conference on Computer Vision.

[139] Ma Q., Li Y., Ren B., Sebe N., Konukoglu E., Gevers T., Van Gool L., Paudel D.P. (2025). A large-scale dataset of gaussian splats and their self-supervised pretraining. 2025 International Conference on 3D Vision (3DV).

